# Improved Neomycin Sulfate Potency in *Streptomyces fradiae* Using Atmospheric and Room Temperature Plasma (ARTP) Mutagenesis and Fermentation Medium Optimization

**DOI:** 10.3390/microorganisms10010094

**Published:** 2022-01-01

**Authors:** Fei Yu, Min Zhang, Junfeng Sun, Fang Wang, Xiangfei Li, Yan Liu, Zhou Wang, Xinrui Zhao, Jianghua Li, Jian Chen, Guocheng Du, Zhenglian Xue

**Affiliations:** 1 Key Laboratory of Industrial Biotechnology, Ministry of Education, School of Biotechnology, Jiangnan University, 1800 Lihu Road, Wuxi 214122, China; 7190201070@stu.jiangnan.edu.cn (F.Y.); 7180201046@stu.jiangnan.edu.cn (X.L.); zhaoxinrui@jiangnan.edu.cn (X.Z.); lijianghua@jiangnan.edu.cn (J.L.); jchen@jiangnan.edu.cn (J.C.); 2 Microorganism Fermentation Engineering and Technology Research Center of Anhui Province, College of Biologic & Food Engineering, Anhui Polytechnic University, 8 Middle Beijing Road, Wuhu 241000, China; zmahpu@163.com (M.Z.); sjf2170420103@163.com (J.S.); wangyw99908@163.com (F.W.); liuyan@ahpu.edu.cn (Y.L.); wangzhou@ahpu.edu.cn (Z.W.); 3 Anhui Engineering Laboratory for Industrial Microbiology Molecular Breeding, Anhui Polytechnic University, 8 Middle Beijing Road, Wuhu 241000, China; 4 Science Center for Future Foods, Jiangnan University, 1800 Lihu Road, Wuxi 214122, China; 5 Key Laboratory of Carbohydrate Chemistry and Biotechnology, Ministry of Education, Jiangnan University, 1800 Lihu Road, Wuxi 214122, China

**Keywords:** neomycin sulfate (NM), *Streptomyces fradiae*, high-throughput screening, atmospheric and room temperature plasma (ARTP) mutagenesis, fermentation medium optimization

## Abstract

To improve the screening efficiency of high-yield neomycin sulfate (NM) *Streptomyces fradiae* strains after mutagenesis, a high-throughput screening method using streptomycin resistance prescreening (8 μg/mL) and a 24-deep well plates/microplate reader (trypan blue spectrophotometry) rescreening strategy was developed. Using this approach, we identified a high-producing NM mutant strain, *Sf*6-2, via six rounds of atmospheric and room temperature plasma (ARTP) mutagenesis and screening. The mutant displayed a NM potency of 7780 ± 110 U/mL and remarkably stable genetic properties over six generations. Furthermore, the key components (soluble starch, peptone, and (NH_4_)_2_SO_4_) affecting NM potency in fermentation medium were selected using Plackett-Burman and optimized by Box-Behnken designs. Finally, the NM potency of *Sf*6-2 was increased to 10,849 ± 141 U/mL at the optimal concentration of each factor (73.98 g/L, 9.23 g/L, and 5.99 g/L, respectively), and it exhibited about a 40% and 100% enhancement when compared with before optimization conditions and the wild-type strain, respectively. In this study, we provide a new *S. fradiae* NM production strategy and generate valuable insights for the breeding and screening of other microorganisms.

## 1. Introduction

Neomycin sulfate (NM, the sulfate salt form of neomycin) was the first 2-deoxystreptamine-containing aminoglycoside antibiotic discovered during *Streptomyces fradiae* fermentation [1]. NM is widely used as a broad spectrum, water soluble antibiotic that inhibits Gram-negative and Gram-positive bacteria. NM also has a narrow therapeutic range due to potential nephrotoxicity and ototoxicity issues, but its use as a treatment for hepatic encephalopathy and hepatocellular carcinoma [2], human immunodeficiency virus [3], human genetic diseases [3], and catheter-associated urinary tract infections [4] has seen demand rapidly increase in recent years.

Natural selection is the main driving force of biological evolution; however, mutation frequencies are typically very low, especially for changes in specific phenotypes [5]. Therefore, artificial random mutation methods, including physical and chemical mutagenesis, comprising alkylating agents (ethyl methane-sulfonate and nitrosoguanidine) [6], azides (sodium and potassium azides) [7], conventional radiation (X-ray and γ-ray) [7], and heavy ion beams [6], have been widely used to increase mutation rates and derive desired phenotypes [8]. However, the tools for these random mutagenesis methods are disadvantageous in terms of poor operability and high safety risks due to chemical and physical mutagens at high toxicity or radiation levels. The atmospheric and room temperature plasma (ARTP) mutagenesis system uses radiofrequency atmospheric-pressure glow discharge (RF-APGD) plasma and acts on organisms under different operating parameters. This incurs DNA damage which is subsequently repaired by the SOS (“Save Our Soul”) system leading to DNA alterations [8,9]. ARTP technology generates high mutation frequencies, is simple and safe, and is widely used for bacteria [10,11,12,13], fungi [14,15], and microalgae [16,17,18]. On the other hand, mutations in strains due to streptomycin resistance are closely related to their ability to produce antibiotics [19]. Specifically, ppGpp (guanosine 5′-diphosphate 3′-diphosphate) accumulation, triggered by stringent responses, is thought to play key roles in antibiotic biosynthesis initiation, whereas mutant strains generating streptomycin resistance allow for antibiotic production/initiation without ppGpp requirements [20,21]. Therefore, introducing mutations conferring streptomycin resistance is widely used in bacteria and *Streptomyces* sp. for improving antibiotic and other metabolite production [22,23,24].

In the early years, metabolite/antibiotic screening was conducted on single colonies, which after physical or chemical mutagenesis, were randomly selected from agar plates, fermented in shake-flask and processed. Such traditional screening methods generated large workloads, were time-consuming, and had low efficiency and high costs, and were gradually eliminated over time. Currently, high-throughput screening technologies based on microplate (24/48/96/384-deep well plates) technologies are widely used for screening mutant strains, biologically active substances, and candidate drugs [25,26,27] due to their similar characteristics to automatic parallel microreactors. In addition, spectrophotometry based microplate readers have huge advantages when compared with cylinder plate and high-performance liquid chromatography (HPLC) methods in terms of antibiotic identification, as they process large numbers of simultaneous samples and reduce reagent costs and use. Therefore, high-throughput screening technologies combined with microplate readers can greatly improve the screening efficiency of mutant strains after mutagenesis.

In this study, we used multiple strategies to improve the screening efficiency of high-producing NM mutant strains. First, we used ARTP technology on wild-type *S. fradiae* to generate large numbers of mutant strains. We then prescreened strains on streptomycin plates using correlations between resistance mutations and antibiotic biosynthesis. Next, prescreened strains were rescreened in 24-deep well fermentation plates and via a newly developed detection method for NM potency (Trypan blue (TB) spectrophotometry based on a microplate reader). The key components in fermentation medium affecting NM potency were selected and optimized using Plackett-Burman (PB) and Box-Behnken designs. Finally, the high-producing NM mutant strain, *Sf*6-2 was identified. Its NM potency was 10,849 ± 141 U/mL, which equated to an enhancement of 40% and 100% when compared with before optimization conditions and the wild-type strain, respectively.

## 2. Materials and Methods

### 2.1. Materials

TB, 9-fluorenylmethoxycarbonyl chloride (FMOC-Cl), and flow phase (acetonitrile) were purchased from Sigma-Aldrich Co., Ltd. (Shanghai, China). NM standards were purchased from XINYU Pharmaceutical Co., Ltd. (642 U/mg; Suzhou, China). All other standard reagents were purchased from Sinopharm Chemical Reagent Co., Ltd. (Shanghai, China). Deep-well plates were purchased from Shanghai Canvic Bio-Technology Co., Ltd. (Shanghai, China). The microplate reader (Multiskan FC) was purchased from Thermo Fisher Scientific (Shanghai, China). The HPLC system (LC-2010HT) was purchased from Shimadzu (Shanghai, China).

### 2.2. Strains and Media

*S. fradiae* GC 6010 (wild-type strain and stored in our laboratory) and 300 mutant strains were generated by ARTP mutagenesis. Strains were grown on solid medium (glucose 10 g, beef extract 1 g, peptone 3 g, corn steep liquor 3 g, NaCl 5 g, and agar 20 g dissolved in 1 L deionized water; pH 7.3–7.8) for 7 days at 30 °C. Single colonies were transferred to seeding medium (soluble starch 10 g, peanut meal 10 g, yeast extract 20 g, (NH_4_)_2_SO_4_ 1 g, glucose 30 g, corn steep liquor 10 g, peptone 5 g, Na_2_HPO_4_ 1 g, CaCO_3_ 10 g, and soybean oil 2 g, pH 7.3–7.8; and made up to 1 L in deionized water) and grown in 24-deep well plates (2 mL volume) at 220 rpm and 35 °C for 30 h. Seed cultures (8% inoculum) were then transferred to fermentation medium (soluble starch 70 g, peanut meal 28 g, yeast extract 6 g, (NH_4_)_2_SO_4_ 6 g, glucose 20 g, corn steep liquor 2.5 g, peptone 9 g, soybean meal 5 g, NaCl 4.5 g, and soybean oil 3 g, pH 6.8–7.3; and made up to 1 L in deionized water) in 24-deep well plates (2 mL volume) and grown at 220 rpm and 35 °C for 7 days.

### 2.3. ARTP Mutagenesis and Screening

**Figure 1:**
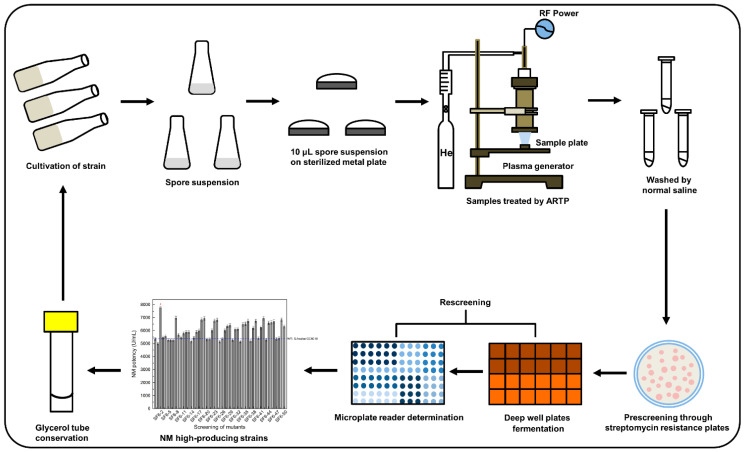
ARTP mutagenesis and screening schematic. RF: radio-frequency.

**Figure 2:**
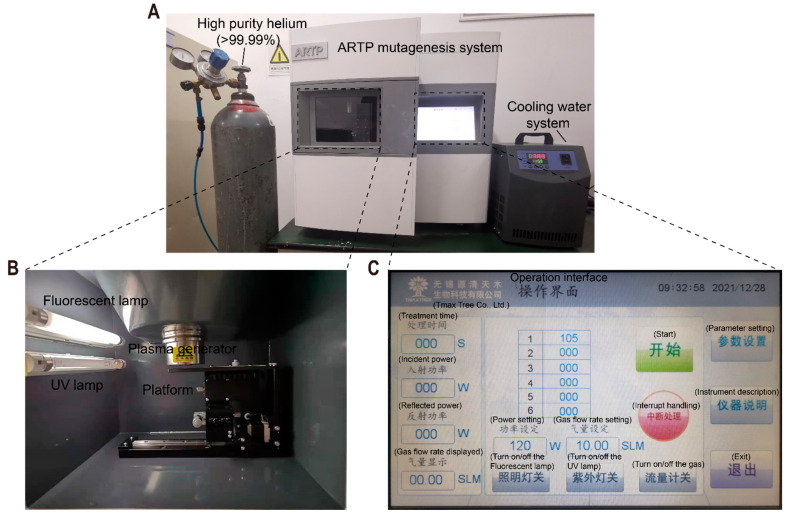
Pictures of the ARTP mutation breeding system. (**A**) The front view; (**B**) The operation chamber; (**C**) The operation interface.

The general workflow involved the preparation of *S. fradiae* spore suspensions followed by ARTP treatment (Si Qing Yuan Biotechnology Co., Ltd./now Tmax Tree Co., Ltd.; Wuxi, China) and pre- and rescreening (Figure 1). The ARTP system (model: ARTP-IIS; weight: 95 kg; voltage: 220 V-50/60 Hz 500 VA; size: 73 cm × 65 cm × 69 cm; Figure 2) needs high-purity helium (>99.99%) as a working gas and a typical electric socket as a power source. Inside the ARTP operation chamber, the helium flowing through the discharge region between the two electrodes is ionized by the radio frequency electric field and then acts on the microbial sample fixed on the metal plate sheet on a regulating platform via the nozzle. Since the breakdown voltage is not high (100–200 V), the plasma maintains discharge consistency, derives little ultraviolet radiation, and combines with the cooling of the cathode to maintain a biocompatible gas temperature. The continuously flowing gas seldom mixes with the surrounding air, thereby minimizing the production of germicidal ozone. It has been reported that the generation of reactive chemical species (He*, He_2_*, He^+^, He_2_^+^, and N_2_^+^) was considered as the biggest cause of physical plasma mutagenesis [28,29], and therefore requires careful adjustment of the plasma-generating parameters. The ARTP manufacturer provides a standard value for each adjustable parameter to meet the conditions of biocompatibility and produce enough active chemical substances [9]. Firstly, it is recommended to use a gas flow rate of 10 SLPM (standard liters per minute) or above to prevent the gas from combining with the surrounding air to produce germicidal ozone. Further research showed that when the gas flow rate is between 5–30 SLPM, the production of active materials is proportional to the gas flow rate [8]. Secondly, the suggested value of the distance between the sample and nozzle is 2 mm, which was used in almost all reports. In line with increasing distance (2–10 mm), the active chemical substances generate decreased sharply and not enough to cause damage to the cells [8]. Thirdly, it was found that the temperature was within a biologically compatible range between 36 °C and 57 °C when the energy was between 40 W and 200 W [9]. In the early study, the radio frequency power input of 40 W was applied in obtaining high-yield butanol *Clostridium acetobutylicum* strain [30]. Finally, for different species, the recommended values for the treatment time are different, including bacteria (15–120 s), *Actinomycetes* (30–180 s), fungi (60–360 s), yeast (30–240 s), and microalgae (5–150 s) [8]. Early research showed that along with the increasing time (0.5–10 min), more DNA damages in cells was produced under the same conditions as other parameters [28].

Based on the analysis of aforementioned adjustable parameters, the following ARTP parameters were used: spore suspension = 10 μL (10^6^–10^8^ cells/mL), helium gas flow rate = 10 SLPM, 2 mm = distance between sample and nozzle, radiofrequency power input = 40 W, and ARTP treatment times = 0–210 s. Untreated spores were used as controls. Several constants were used to evaluate the effects of multiple ARTP mutagenesis rounds, and were calculated based on Equations (1)–(5).

Lethality (%) = (*U − T*)/*U* × 100, (1)

Mutation rate (*R_M_*) (%) = *M*/*T* × 100, (2)

Positive mutation rate (*R_P_*) (%) = *P/M* × 100, (3)

Relative NM potency of mutants (%) = *N*/*W* × 100, (4)

Average relative NM potency of mutants (%) = *A*/*W* × 100, (5)

where *U* = total colony counts in untreated samples, *T* = total number of colonies after ARTP treatment, *M* = total colony count of mutant strains which the NM potency different from wild-type strain (the difference was above ±2%), *P* = total colony counts of mutant strains with higher NM potencies than the wild-type strain (>2%), *N* = NM potency of mutant strains in each mutagenesis round, *W* = NM potency of the wild-type strain (*S. fradiae* GC 6010), and *A* = average NM potency of mutant strains generated in each mutagenesis round.

Then, treated spore suspensions were resuspended in 0.9% NaCl, diluted, and spread onto streptomycin agar plates for prescreening. The concentrations of streptomycin-resistant screening plates were 0, 2, 4, 6, 8, and 10 μg/mL. Plates without streptomycin were used as controls.

Next, single colonies after prescreening were transferred to seeding medium in deep-well plates. Then, seed cultures were transferred to a fermentation medium in deep-well plates. We used 24-deep well plates, 48-deep well plates, and 250 mL shake flask for fermentation, with fermentation correlations used for analysis by fitting data into Origin 9 software.

### 2.4. Method Development to Assess NM Potency in Fermentation Broth

Fermented media (7 days, 220 rpm, 35 °C) was centrifuged for 10 min at 10,000 rpm and supernatants were collected to determine NM potency. When TB reacts with NM, ions become associated and a blue color is formed [31]. At a particular wavelength, the NM potency in fermentation broth could be determined using a microplate reader.

#### 2.4.1. Selection of the Detection Wavelength

A 100 μL NM standard solution (25.68 U/mL) was mixed with 100 μL Britton-Robison buffer (pH 6.5), then 300 μL TB solution (1.0 × 10^−4^ mol/L) added and made up to 1 mL with deionized water. The reaction was incubated at room temperature for 10 min. Finally, the solution underwent a full-wavelength scan in a microplate reader to determine the maximum absorption wavelength peak. As a control, the TB solution was replaced with deionized water.

#### 2.4.2. Optimizing TB Solution (1.0 × 10^−4^ mol/L) Volumes

Different volumes (50, 100, 150, 200, 250, 300, 350, and 400 μL) of the NM standard solution (25.68 U/mL) were mixed with 100 μL Britton-Robison buffer (pH 6.5), and then different volumes (100, 200, 300, 400, and 500 μL) of TB solution (1.0 × 10^−4^ mol/L) were added in separate experiments. Deionized water was then added to 1 mL and the reaction was incubated at room temperature for 10 min. Finally, the absorbance of different solutions was determined at the maximum absorption wavelength to determine the optimal TB solution (1.0 × 10^−4^ mol/L) volume. A standard curve of NM potency versus absorbance was generated.

#### 2.4.3. Spike and Recovery Studies

We divided a fermentation broth of known NM potency into five parts and added NM standard solutions (25.68 U/mL) of different volumes (75, 125, 175, 225, 275, 325, and 375 μL). The NM potency in different solutions was determined by the aforementioned optimized method, and average recovery rates, with relative standard deviation (*RSD*), were calculated based on Equations (6)–(10).

NM standard addition amount (*A_S_*) (μg) = (*V*_1_ × *S*_1_)/*D*, (6)

Measured amount (*A_M_*) (μg) = (*V*_2_ × *d_S_*)/*D*, (7)

Recovery rate (%) = *A_M_*/*A_S_* × 100, (8)

Average recovery rate (*X*) (%) = *E*/*n*, (9)

*RSD* (%) = *SD*/*X* × 100, (10)

where *V*_1_ = NM standard solution volume, *S*_1_ = potency of the NM standard solution (25.68 U/mL), *D* = NM standard potency (642 U/mg), *V*_2_ = volume of the reaction system (1 mL), *d_S_* = the measured NM potency minus the NM potency of the known fermentation broth in the reaction system, *E* = sum of all recovery rates, *n* = number of spike and recovery experiments, *X* = average recovery rate, and *SD* = standard deviation of all recovery rates.

### 2.5. Correlation Analyses between the New Method and HPLC

The 25 mutant strains generated after ARTP mutagenesis were inoculated and fermented in deep well plates. Then, the newly established method and HPLC were simultaneously compared for NM potency assessment, with data fitting analysis performed using Origin 9 software. The following HPLC steps were performed [1,32]: a mixture of 1 mL fermentation supernatant, 1 mL acetonitrile, and 1 mL borax-boric acid buffer (0.2 mol/L, pH = 8.0) were added to 2 mL FMOC-Cl solution (8 mmol/L; made with acetonitrile) at 25 °C and lightly agitated for 15 min in the dark. The reaction was stopped by adding a 200 μL glycine solution (0.1 mol/L). The solution was filtered through a 0.22 μm syringe filter and subjected to reversed-phase HPLC analysis to assess NM potency. The following HPLC parameters were used: chromatographic Agilent C18 column (150 mm × 4.6 mm, 5 μm), flow rate = 1 mL/min, flow phase of acetonitrile/water (95:5, *v*/*v*), column temperature = 25 °C, and injection volume = 10 μL. Absorption was monitored at 265 nm. The retention time for NM was approximately 5.5–6.0 min under these analytical conditions.

### 2.6. Optimization of Fermentation Medium

At first, PB designs were used to screen for key components in the fermentation medium affecting NM potency. In total, 10 components were selected, and it was assumed no interactions occurred between them. Based on PB regression analysis, components with significant *p* < 0.05) values were selected for further optimization.

Interactions between these significant factors were investigated using Box-Behnken designs. Next, a second-order polynomial equation was obtained using Design-Expert 8.0.2 software based on analysis of variance (ANOVA).

(11) $$Y=\alpha_{0}+\sum \alpha_{i}X_{i}+\sum \alpha_{ii}X_{i}^{2}+\sum \alpha_{ij}X_{i}X_{j},$$

where *Y* = the predicted response of NM potency; $\alpha_{0\;}$ = the value of the fitted response at the center point of the design; $\alpha_{i}$, $\alpha_{ii}$, and $\alpha_{ij}$ = linear, quadratic, and cross-product regression terms, respectively; $X_{i}$ = independent variables.

Then, optimal values for independent variables and corresponding predicted responses for NM potency were calculated using the second-order polynomial equation. Finally, fermentation was conducted using these optimal independent variables to verify the accuracy of the predicted response.

## 3. Results

### 3.1. Establishing a Prescreening Method for Mutant Strains

Currently, ribosome engineering using resistance mutations in microorganisms as screening markers is a new breeding method that generates mutant strains with an improved ability to synthesize secondary metabolites [33,34]. Streptomycin resistance mutations are the most frequently used for screening high-yielding antibiotic strains, including actinorhodin (48-fold) in *Streptomyces coelicolor* A3(2) [35], fredericamycin (26-fold) in *Streptomyces chattanoogensis* [19], and salinomycin (2.3-fold) in *Streptomyces albus* [36].

**Figure 3:**
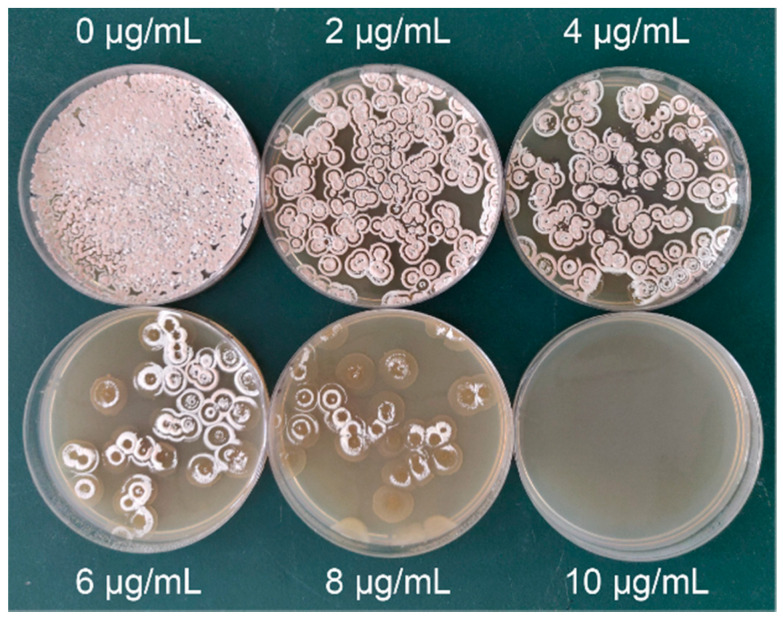
Prescreening mutant strains on resistant plates containing different streptomycin concentrations (0, 2, 4, 6, 8, and 10 μg/mL).

Therefore, our mutant strains, produced after ARTP mutagenesis, were prescreened using streptomycin plates; however, starting streptomycin concentrations for plates had to be chosen. In line with increasing concentrations in resistance plates (0–8 μg/mL), colony numbers decreased sharply (Figure 3) and more improved NM potency mutant strains were generated (Table 1). When the streptomycin concentration was 10 μg/mL, all strains died (Figure 3). Therefore, the streptomycin concentration for prescreening resistance plates was 8 μg/mL.

### 3.2. Establishing a Rescreening Method for Mutant Strains

#### 3.2.1. Selecting Deep-Well Plates for Mutant Strain Fermentation

**Figure 4:**
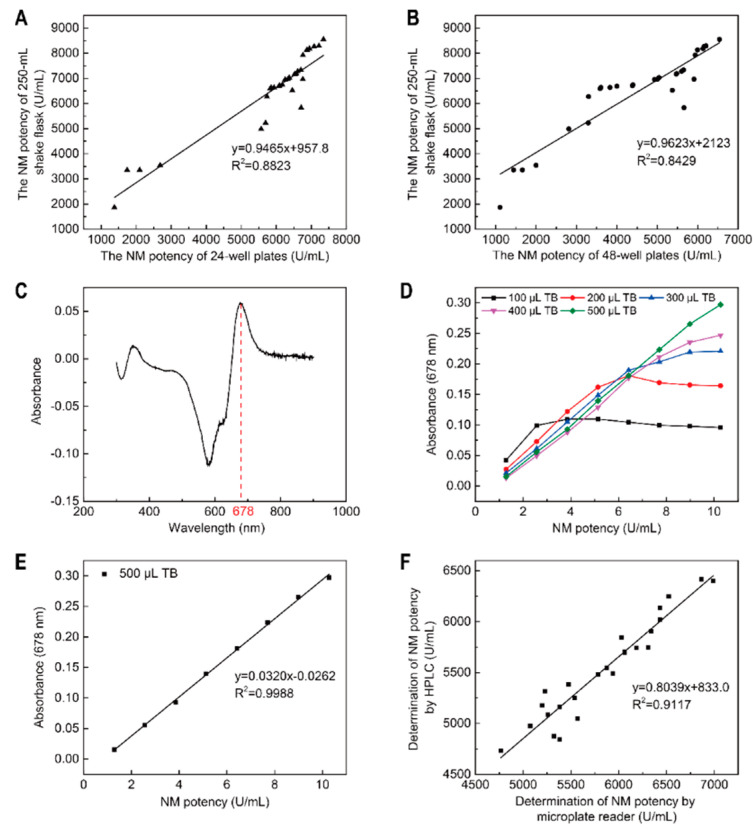
Optimization of the rescreening method for high-producing NM mutant strains. (**A**) Fermentation correlations between 24-deep well plates and 250 mL shake flasks; (**B**) Fermentation correlations between 48-deep well plates and 250 mL shake flasks; (**C**) A full-wavelength scan of TB and NM reaction solutions; (**D**) The influence of different TB volumes (1.0 × 10^−4^ mol/L) on the absorbance at different NM standard potencies; (**E**) A NM standard curve; (**F**) Correlations between TB spectrophotometry using a microplate reader and HPLC for NM potency determination.

When compared with shake flasks, deep well plates are more suitable for high-throughput mutant strain screening. However, it was unclear which plates had similar fermentation effects as shake flasks, therefore an investigation was conducted. We investigated correlations between different plates (24/48-deep well plates) and 250 mL shake flask fermentations. We showed that the correlation between 24-deep well plates and 250 mL shake flask fermentation was higher than 48-deep well plates with 250 mL shake flasks (*R*^2^_24-well plates_ = 0.8823 > *R*^2^_48-well plates_ = 0.8429; Figure 4A,B). Thus, 24-deep well plates were better alternatives to shake flasks and were ideal for the rapid rescreening of large numbers of mutant strains after prescreening.

#### 3.2.2. Developing a New Method to Assess NM Potency in Fermentation Broth

Currently, cylinder plate and HPLC methods are routinely used to determine antibiotic potency [37,38,39], but they are unsuitable for high-throughput mutant strain screening. To address this, we established a method to rapidly determine NM potency in the fermentation broth. TB is an azo dye that reacts with NM to induce a color change and obvious absorption peaks [31]. Therefore, TB was used to detect NM potency in the fermentation broth. The blue complex formed by the NM/TB reaction generated a maximum absorption peak at 678 nm using full-wavelength scanning on a microplate reader (Figure 4C).

Next, we assessed different volumes of TB solution (1.0 × 10^−4^ mol/L) on absorbance at different NM standard potency levels. When the NM potency standard was between 1.284 and 10.272 U/mL, and the volume of the TB solution (1.0 × 10^−4^ mol/L) was between 100 and 400 μL, NM potency and absorbance were not linear (Figure 4D). This suggested that the TB volume was inadequate for NM and TB to form a blue complex. Therefore, the volume of TB solution (1.0 × 10^−4^ mol/L) was increased; at 500 μL, the absorbance displayed a good linear correlation with the NM potency and a good linear equation was obtained (Figure 4D,E).

*Y* = 0.0320 *x* − 0.0262 (*R*^2^ = 0.9988), (12)

Furthermore, spike-and-recovery experiments were conducted to verify method accuracy. These showed an average recovery rate of 98.4684% and *RSD* = 3.2700% between low level (3 μg NM standard) and high level (15 μg NM standard) standards (Table 2). Thus, our method was highly accurate for detecting NM potency in the fermentation broth.

Finally, we used this new method and HPLC to simultaneously detect NM potencies of 25 mutant strains fermented in 24-deep well plates. These data indicated a high correlation coefficient (*R*^2^ = 0.9117) between this method and HPLC (Figure 4F), which suggested the new method could replace HPLC. Additionally, the new method was not only rapid (10 min) but simultaneously determined NM potencies in many samples. Therefore, this TB spectrophotometry-based microplate reader method was suitable for the high-throughput screening of mutant strains.

### 3.3. Iterative ARTP Mutagenesis and Screening

**Figure 5:**
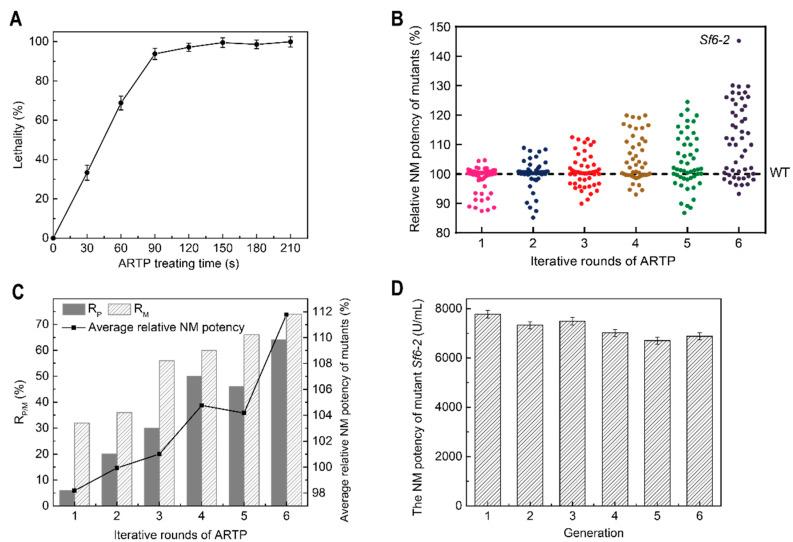
Iterative ARTP mutagenesis and investigations of genetic stability. (**A**) A *S. fradiae* lethality plot by ARTP mutagenesis; (**B**) The distribution of mutant strains with different NM potencies in each ARTP round; (**C**) The cumulative effects of iterative ARTP mutagenesis on NM potency in mutant strains; (**D**) Genetic stability of the high-producing NM mutant strain, *Sf*6-2. WT: wild-type strain (*S. fradiae* GC 6010); *R_P_*: positive mutation rate; *R_M_*: mutation rate; *R_P/M_*: *R_P_* and *R_M_*.

When compared with other breeding methods, ARTP has a higher mutation frequency [9]. Before performing continuous ARTP mutagenesis and screening, the effects of different ARTP treatment times on strain growth were investigated as survival rates and mutation frequencies can be significantly affected by implanted doses [40]. At first, strain lethality was gradually increased when treatment times increased from 0 to 150 s (Figure 5A). Then, lethality slightly declined when the time ranged from 150 to 180 s (Figure 5A). This phenomenon was explained by the repair mechanism inside the cell (SOS) becoming activated under corresponding ion dose ranges [41,42]. After this, more mutant strains were produced. When treatment times exceeded 180 s, self-repair mechanisms lagged behind cellular damage, and therefore the strain was practically dead [40]. Thus, 180 s, which corresponded to the optimal ion dosage, was chosen for mutation induction.

After six rounds of ARTP mutagenesis and screening (the top four mutant strains with the highest NM potency in each round were chosen as starting strains for the next), a high-producing NM strain, *Sf*6-2 was finally screened out from 300 prescreened strains. It displayed a higher NM potency at 45% more than the wild-type strain (*S. fradiae* GC6010) and generated 7780 ± 110 U/mL (Figure 5B). Furthermore, the cumulative effects of multiple rounds of ARTP mutagenesis on NM potency in mutants were investigated. As the number of iterations increased, the proportion of mutant strains gradually increased, with the proportion of positive mutant strains showing an increasing trend (Figure 5B,C). Thus, multiple ARTP mutagenesis rounds still generated good cumulative effects in mutants for NM production. We also assessed the genetic stability of *Sf*6-2 for industrial production; the mutant displayed remarkably stable NM production levels over six generations (Figure 5D).

### 3.4. Optimization of the Fermentation Medium

As the growth properties of mutant strains may have changed, and the original fermentation medium was no longer suitable for target product synthesis, it therefore required optimization. We used PB designs (Table 3) and regression analysis (Table 4) to screen for the three key components vital for NM potency in *Sf*6-2 from the original fermentation medium. These were soluble starch, peptone, and (NH_4_)_2_SO_4_ (*p*-value < 0.05). Response coefficients also indicated that these variables had huge effects on NM potency (Table 4) [43].

**Figure 6:**
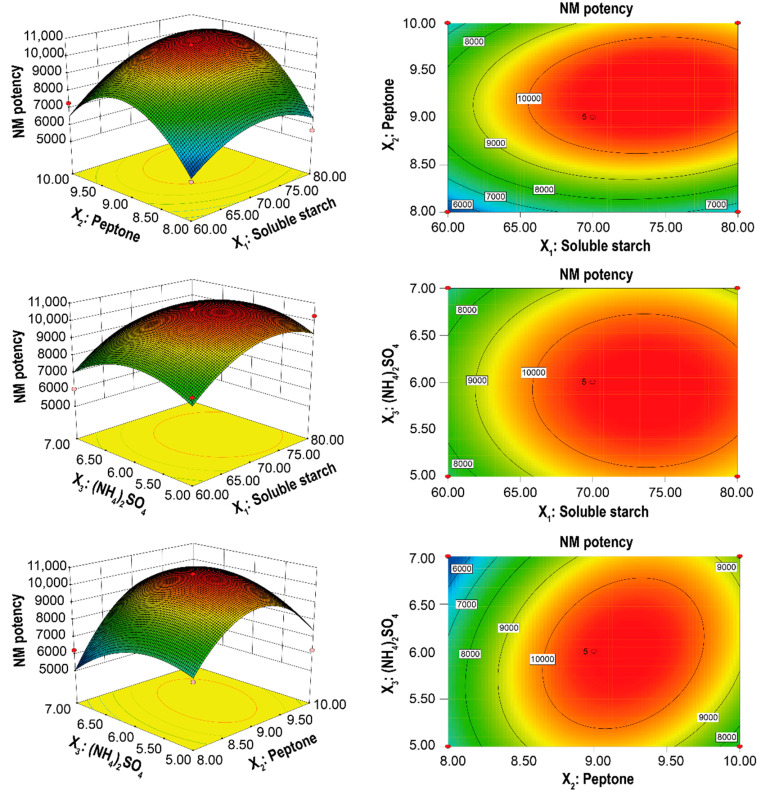
Response surface plots showing the effects of (*X*_1_) soluble starch, (*X*_2_) peptone, and (*X*_3_) (NH_4_)_2_SO_4_ on NM potency.

These factors were then optimized by Box-Behnken design (Table 5 and Table 6) and ANOVA analysis (Table 7). Model precision and reliability were both demonstrated by the correlation coefficient, *R*^2^ = 0.9026, and the coefficient of variation = 11.57%. The adjusted *R*^2^ = 0.78 was also close to the actual *R*^2^, indicating predictive model responses were adequate. In addition, the *Prob* > *F* (*p*-value) of the model was calculated as 0.0082 < 0.01, meaning the regression model was extremely significant. Next, the interaction of these factors was assessed. According to regression analysis of the experimental design, the interaction terms (*X*_1×2_, *X*_1×3_, and *X*_2×3_) were not significant (*p*-value > 0.05), while the linear terms (*X*_1_ and *X*_2_) and second order terms (*X*_1_^2^, *X*_2_^2^, and *X*_3_^2^) were significant, especially for the *X*_2_^2^ s order term (*p*-value < 0.01). On the other hand, a three-dimensional view (Figure 6) displayed clearly in a contour plot of the response surface and showed that along with the increasing soluble starch, peptone, and (NH_4_)_2_SO_4_, the NM potency was improved and reached to maximum at the optimal condition of the three factors. However, NM potency decreased sharply with increases in the three components in the fermentation medium.

Furthermore, a second-order polynomial equation, which reflected the impact of all terms (linear, quadratic, and interactive) on the response appropriately, was used to predict the NM potency of *Sf*6-2 after fermentation:

*Y* = 10,608.00 + 934.13 × *X*_1_ + 988.50 × *X*_2_ − 194.63 × *X*_3_ + 404.75 × *X*_1_ × *X*_2_ − 31.00 × *X*_1_ × *X*_3_ + 762.75 × *X*_2_ × *X*_3_* − *1295.00 × *X*_1_^2^
* − * 2431.75 × *X*_2_^2^ − 1186.50 × *X*_3_^2^, (13)

The optimal concentration of each factor (soluble starch, peptone, and (NH_4_)_2_SO_4_) was generated using this second-order polynomial equation; concentrations were 73.98 g/L, 9.23 g/L, and 5.99 g/L, respectively, and the corresponding NM potency of *Sf*6-2 was 10,910 U/mL. Finally, *Sf*6-2 was fermented under these optimal conditions to verify model validity. This generated an NM potency (practical response) of 10,849 ± 141 U/mL, which was approximately 99% of the predicted value and indicated perfect agreement with the model. After this optimization, the actual NM potency of *Sf*6-2 displayed an enhancement of 40% and 100% when compared to before optimization conditions and the original strain, respectively.

## 4. Discussion

We reported a rapid screening method for high−producing NM mutant strains. The process consisted of three modules: a commercially available ARTP mutagenesis system, prescreening on streptomycin plates, and rescreening using 24−deep well plates, and a new detection method (TB spectrophotometry using a microplate reader). This approach had multiple advantages: (1) the ARTP mutation system is superior to other conventional methods in some respects [8,9]. When generating mutant strains, costs are greatly reduced and safety is considerably improved when compared with traditional physical and chemical mutagenesis. In addition, more high frequency random mutations, induced by reactive chemical species produced by the helium−based ARTP, could generate desirable stable genetic phenotypes in a simple to operate manner. (2) Using streptomycin plates for prescreening not only improved the screening efficiency, but also triggered *S. fradiae* to produce more NM. Currently, the application of streptomycin resistance has improved the biosynthesis and growth tolerance properties of diverse bacterial and fungal species [22,23,24]. (3) Rescreening based on 24−deep well plates and TB spectrophotometry using a microplate reader greatly improved screening efficiency when compared with traditional screening methods (shake-flask fermentation combined with HPLC determination). These advantages meant this method was well suited for high-producing NM mutant strain screening.

After six rounds of breeding and screening, a high−producing NM mutant strain, *Sf*6-2 was identified and displayed an NM potency of 7780 ± 110 U/mL. This equated to an increase of 45% when compared with the wild−type strain. In addition, these data demonstrated ARTP feasibility in generating high−yielding NM mutant strains. For mutant strains generated by iterative mutagenesis, typically the original fermentation medium is no longer suitable for product synthesis, and further component optimization is required. Therefore, key fermentation media factors (soluble starch, peptone, and (NH_4_)_2_SO_4_) which impacted NM potency were screened using PB designs. Subsequently, these components were further optimized using Box-Behnken designs and used at the optimized concentrations of 73.98 g/L, 9.23 g/L, and 5.99 g/L, respectively. Finally, the NM potency of *Sf*6-2 reached 10,849 ± 141 U/mL and reflected an increase of 40% when compared with before optimization conditions, and a two-fold increase on the wild−type strain.

**Figure 7:**
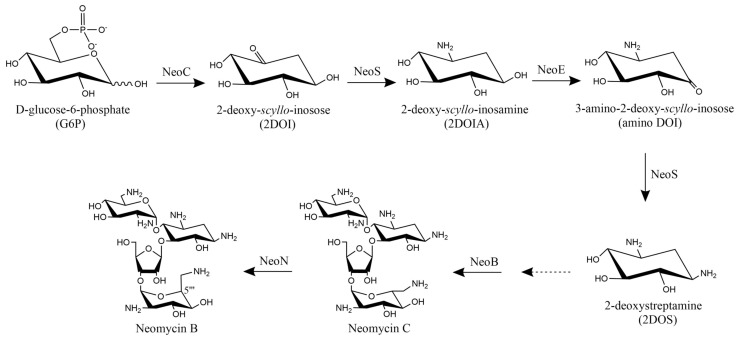
The biosynthetic pathway of neomycin B. NeoC: 2-deoxy-*scyllo*-inosose (2DOI) synthase; NeoS: 2DOI aminotransferase; NeoE: 2-deoxy-*scyllo*-inosamine dehydrogenase; NeoB: 6′-oxoparomamine aminotransferase; NeoN: neomycin C 5‴-epimerase.

In the future, three possible research directions can be taken to improve *S. fradiae* NM potency: (1) The overexpression of neomycin C 5‴-epimerase (NeoN) which is involved in the last step of NM biosynthesis [44]. The last step of NM biosynthesis is the epimerization of neomycin C to neomycin B (the main component of NM); thus neomycin C is generally considered the biosynthetic precursor of neomycin B (Figure 7). It was previously reported that epimerization was the reason why the antibacterial activity of neomycin C was lower than neomycin B, and thus, epimerization at C-5‴ of neomycin C may be a rate-limiting step in the entire biosynthetic pathway [45]. Therefore, *S. fradiae* NM potency may be greatly improved by overexpressing NeoN. (2) Transcriptomic analysis could be conducted on the wild-type *S. fradiae* GC 6010 strain and *Sf*6-2 to identify and classify differentially expressed genes into related metabolic pathways. This approach could identify possible mechanisms underpinning increased NM potency in *Sf*6-2. (3) Fermentation broth viscosity could be optimized as it affects antibiotic production [46,47], but has received little research attention. By optimizing carbon and nitrogen types and proportions, fermentation broth viscosity, suitable for NM production, could be optimized to further increase NM potency.

## Figures and Tables

**Table 1:** The distribution fraction of mutant strains on streptomycin resistance plates.

The Concentrations of Streptomycin (μg/mL)	Distribution Fraction of Mutant Strains with Different NM Potency (%)			
	<6000 *	6000–6500 *	6500–7000 *	>7000 *
2	49.6	42.3	8.1	0
4	32.6	44.4	20.5	2.5
6	22.6	30.5	38.6	8.3
8	12.1	22.4	48.7	16.8

The 80, 60, 30, and 20 mutant strains were randomly selected from 2, 4, 6, and 8 μg/mL streptomycin plates, respectively, and underwent 24-deep well plates fermentation and further NM potency assessment. * Represents NM potency (U/mL).

**Table 2:** Spike and recovery experiments.

Addition Amount (μg)	Measured Amount (μg)	Recovery Rate (%)	Average Recovery Rate (%)	*RSD*/%
3	2.8943 ± 0.0289	96.4770	98.4684	3.2700
5	4.7008 ± 0.0564	94.0163		
7	6.6878 ± 0.0535	95.5401		
9	9.0537 ± 0.1358	100.5962		
11	11.1122 ± 0.2224	101.0200		
13	13.3626 ± 0.2138	102.7892		
15	14.8260 ± 0.3707	98.8401		

The experiments were performed in triplicate. *RSD*: relative standard deviation.

**Table 3:** Plackett-Burman design matrix with corresponding results. *X*_1_–*X*_10_: independent variables; *D*_1_–*D*_4_: dummy variables; (+): high level; (−): low level.

Trial No.	Variables														NM Potency (U/mL)
	*X*_1_	*D*_1_	*X*_2_	*D*_2_	*X*_3_	*D*_3_	*X*_4_	*X*_5_	*X*_6_	*X_7_*	*X*_8_	*X*_9_	*X*_10_	*D*_4_	
1	+	−	+	−	+	+	+	+	−	−	+	+	−	+	8912 ± 107
2	+	+	−	+	+	−	−	−	−	+	−	+	−	+	8284 ± 116
3	+	−	+	+	−	−	−	−	+	−	+	−	+	+	7594 ± 100
4	+	−	+	+	+	+	−	−	+	+	−	+	+	−	7280 ± 87
5	−	−	−	−	+	−	+	−	+	+	+	+	−	−	7782 ± 111
6	+	+	+	+	−	−	+	+	−	+	+	−	−	−	6652 ± 80
7	−	−	+	−	+	−	+	+	+	+	−	−	+	+	8849 ± 115
8	+	+	−	−	−	−	+	−	+	−	+	+	+	+	8033 ± 72
9	−	+	+	−	−	−	−	+	−	+	−	+	+	+	6088 ± 60
10	−	+	+	+	+	−	−	+	+	−	+	+	−	−	7656 ± 97
11	−	+	+	−	+	+	−	−	−	−	+	−	+	−	9602 ± 108
12	−	+	−	+	−	+	+	+	+	−	−	+	+	−	6903 ± 76
13	+	+	+	−	−	+	+	−	+	+	−	−	−	−	9037 ± 80
14	−	−	−	+	−	+	−	+	+	+	+	−	−	+	5962 ± 48
15	−	−	−	−	−	−	−	−	−	−	−	−	−	−	6150 ± 80
16	+	−	−	−	−	+	−	+	−	+	+	+	+	−	4958 ± 55
17	+	−	−	+	+	−	+	+	−	−	−	−	+	−	6401 ± 78
18	−	−	+	+	−	+	+	−	−	−	−	+	−	+	9163 ± 91
19	+	+	−	−	+	+	−	+	+	−	−	−	−	+	8661 ± 105
20	−	+	−	+	+	+	+	−	−	+	+	−	+	+	8033 ± 88

*X*_1_: NaCl; *X*_2_: (NH4)_2_SO_4_; *X*_3_: peptone; *X*_4_: peanut meal; *X*_5_: soluble starch; *X*_6_: soybean meal; *X*_7_: glucose; *X*_8_: soybean oil; *X*_9_: corn steep liquor; *X*_10_: yeast extract.

**Table 4:** Plackett−Burman regression analysis.

Variables	Terms	Values		Coefficient	*t*-Value	*p*-Value	Confidence Level (%)
	Components (g/L)	Low (−)	High (+)				
*X*_1_	NaCl	3.5	5.5	−38	−0.13	0.902	9.8
*X*_2_	(NH_4_)_2_SO_4_	5.0	7.0	967	3.31	0.021	97.9
*X*_3_	Peptone	8.0	10	1092	3.74	0.013	98.7
*X*_4_	Peanut meal	18	38	753	2.58	0.052	94.8
*X*_5_	Soluble starch	60	80	−992	−3.40	0.019	98.1
*X*_6_	Soybean meal	4.0	6.0	351	1.21	0.282	71.8
*X*_7_	Glucose	10	30	−615	−2.11	0.089	91.1
*X*_8_	Soybean oil	2.0	4.0	−163	−0.56	0.600	40.0
*X*_9_	Corn steep liquor	1.5	3.5	−188	−0.65	0.547	45.3
*X*_10_	Yeast extract	5.0	7.0	−452	−1.55	0.182	81.8

**Table 5:** Design of factors and levels.

Values	*X*_1_ (Soluble Starch) (g/L)	*X*_2_ (Peptone) (g/L)	*X*_3_ ((NH_4_)_2_SO_4_) (g/L)
−1	60	8	5
0	70	9	6
1	80	10	7

**Table 6:** Response surface method (RSM) arrangements and results.

Trial No.	*X*_1_ (Soluble Starch)	*X*_2_ (Peptone)	*X*_3_ ((NH_4_)_2_SO_4_)	NM Potency (U/mL)
1	−1	−1	0	5169 ± 62
2	1	1	0	9403 ± 132
3	0	−1	−1	6725 ± 89
4	−1	1	0	7286 ± 87
5	0	0	0	10,611 ± 153
6	0	1	−1	6227 ± 75
7	0	0	0	10,608 ± 138
8	−1	0	1	6040 ± 54
9	0	1	1	8780 ± 86
10	1	−1	0	5667 ± 72
11	−1	0	−1	7784 ± 87
12	0	0	0	10,602 ± 116
13	1	0	1	8407 ± 75
14	0	−1	1	6227 ± 50
15	1	0	−1	10,275 ± 134
16	0	0	0	10,611 ± 118
17	0	0	0	10,608 ± 105

**Table 7:** ANOVA for Box-Behnken design.

Source	Sum of Squares	df	Mean Square	*F*-Value	*Prob* > *F*	Significance
Model	5.976 × 10^7^	9	6.640 × 10^6^	7.21	0.0082	**
*X*_1_	6.981 × 10^6^	1	6.981 × 10^6^	7.58	0.0284	*
*X*_2_	7.817 × 10^6^	1	7.817 × 10^6^	8.49	0.0225	*
*X*_3_	3.030 × 10^5^	1	3.030 × 10^5^	0.33	0.5842	
*X*_1 *×* 2_	6.553 × 10^5^	1	6.553 × 10^5^	0.71	0.4268	
*X*_1 *×* 3_	3844.00	1	3844.00	4.174 × 10^3^	0.9503	
*X*_2 *×* 3_	2.327 × 10^6^	1	2.327 × 10^6^	2.53	0.1559	
*X*_1_^2^	7.061 × 10^6^	1	7.061 × 10^6^	7.67	0.0277	*
*X*_2_^2^	2.490 × 10^7^	1	2.490 × 10^7^	27.04	0.0013	**
*X*_3_^2^	5.928 × 10^6^	1	5.928 × 10^6^	6.44	0.0388	*
Lack of Fit	6.446 × 10^6^	3	2.149 × 10^6^	1.592 × 10^5^	0.0527	
Pure Error	54.00	4	13.50			
Total	6.621 × 10^7^	16				
*C.V.*%	11.57					
*R* ^2^	0.9026					
Adjusted *R*^2^	0.7774					

* statistically significant at 95% of confidence level; ** statistically significant at 99% of confidence level.

## Abbreviations

NM: neomycin sulfate; ARTP: atmospheric and room temperature plasma; RF−APGD: radiofrequency atmospheric−pressure glow discharge; SOS: Save Our Soul; ppGpp: guanosine 5´−diphosphate 3´−diphosphate; HPLC: high−performance liquid chromatography; TB: trypan blue; PB: Plackett−Burman; FMOC−Cl: 9−fluorenylmethoxycarbonyl chloride; SLPM: standard liters per minute; *R_M_*: mutation rate; *R_P_*: positive mutation rate; *RSD*: relative standard deviation; *A_S_*: NM standard addition amount; *A_M_*: measured amount; *SD*: standard deviation; ANOVA: analysis of variance; NeoN: neomycin C 5‴-epimerase.
